# An investigation of sound levels on intensive care units with reference to the WHO guidelines

**DOI:** 10.1186/cc12870

**Published:** 2013-09-03

**Authors:** Julie L Darbyshire, J Duncan Young

**Affiliations:** 1 Nuffield Division of Anaesthetics, Nuffield Department of Clinical Neurosciences, University of Oxford, John Radcliffe Hospital, Headley Way, Oxford, OX3 9DU, UK; 2 Adult Intensive Care Unit, Oxford University Hospitals NHS Trust, John Radcliffe Hospital, Headley Way, Headington, Oxford, OX3 9DU, UK

## Abstract

**Introduction:**

Patients in intensive care units (ICUs) suffer from sleep deprivation arising from nursing interventions and ambient noise. This may exacerbate confusion and ICU-related delirium. The World Health Organization (WHO) suggests that average hospital sound levels should not exceed 35 dB with a maximum of 40 dB overnight. We monitored five ICUs to check compliance with these guidelines.

**Methods:**

Sound levels were recorded in five adult ICUs in the UK. Two sound level monitors recorded concurrently for 24 hours at the ICU central stations and adjacent to patients. Sample values to determine levels generated by equipment and external noise were also recorded in an empty ICU side room.

**Results:**

Average sound levels always exceeded 45 dBA and for 50% of the time exceeded between 52 and 59 dBA in individual ICUs. There was diurnal variation with values decreasing after evening handovers to an overnight average minimum of 51 dBA at 4 AM. Peaks above 85 dBA occurred at all sites, up to 16 times per hour overnight and more frequently during the day. WHO guidelines on sound levels could be only achieved in a side room by switching all equipment off.

**Conclusion:**

All ICUs had sound levels greater than WHO recommendations, but the WHO recommended levels are so low they are not achievable in an ICU. Levels adjacent to patients are higher than those recorded at central stations. Unit-wide noise reduction programmes or mechanical means of isolating patients from ambient noise, such as earplugs, should be considered.

## Introduction

Over 30% of patients treated in ICUs become confused or develop delirium. These patients have longer hospital stays and higher mortality and morbidity [1]. Risk factors for the development of ICU-related delirium are sedation use and invasive procedures, but there also is a link with environmental factors, including noise-induced sleep disturbance [2].

Although there is wide variation due to individual sensitivity to noise and tendency to aggravation [3], the normal healthy adult can tolerate an A-weighted sound level in decibels (dBA) of about 50 to 55 dBA relatively well during the day and 40 to 45 dBA overnight [4]. At these levels, most individuals would not experience annoyance, sleep disturbance or any detrimental health effects. However, quantifiable effects from sleep disturbance can be seen at time-averaged sound levels (L_Aeq_) as low as 30 dBA and corresponding peak noise levels (L_Amax_) of 45 dBA or less [4].

The World Health Organisation (WHO) *Guidelines for Community Noise *includes advice on noise levels in hospitals and suggests that, because patients are less able to cope with the increased stress levels generated by excess environmental noise, the sound level in hospitals should not exceed 35 dBA L_Aeq _for areas where patients are treated or observed, with a corresponding L_Amax _of 40 dBA [4].

We decided to assess the sound levels in the ICUs in our hospital group and adjacent hospitals to see how they compared with these standards.

## Methods

This study was undertaken over two weeks in June 2012. Daytime and night-time sound levels were monitored during the week and at the weekend in five ICUs in the Thames Valley region of England (John Radcliffe Hospital, Oxford, Adult ICU and Neurosciences ICU; Churchill Hospital, Oxford, Adult ICU; Royal Berkshire Hospital, Reading, Adult ICU; and Wycombe General Hospital, High Wycombe, Adult ICU). These units were chosen for both their proximity and their heterogeneity because they are examples of different physical ward layouts, patient populations and building designs. This exercise did not involve patient recruitment or the use of any identifiable information. Our local ethics policy states that studies based on fully anonymised data which the study team cannot trace back to individuals does not constitute 'research involving human participants', and therefore this study was not subject to ethical review. The lead physician at each unit gave written permission for the sound levels to be measured, and staff members working on the units were aware of the monitoring.

Sound levels were collected using a pair of CEL-630 portable sound level monitors with integral recording fitted with a CEL-495 preamplifier and a CEL-251 microphone (Casella Measurement, Kempston, UK). In each ICU, the two monitors ran concurrently for 24 hours for each period of recording, one placed centrally in the unit on or adjacent to the central station and one placed adjacent to a patient's head. Where possible, patients centrally located within their section of the unit were chosen. The devices were calibrated using an acoustic calibrator (CEL-120/1; Casella Measurement) and set to record continuously for 24 hours. Every minute, the devices recorded peak noise levels (L_Apeak_) and an averaged sound level (L_Aeq_) from the preceding 60 seconds. The reference level (0 dB) was the limit of human hearing at a frequency of 1 kHz.

Short-duration sample recordings were also taken in an unoccupied ICU side room in the John Radcliffe Hospital Adult Intensive Care Unit with the monitors and other device alarms sequentially activated. These included examples of the most common alarm signals (Philips IntelliVue MP70 patient monitor, Philips Healthcare, Best, The Netherlands; Puritan Bennett 840 ventilator, Covidien, Dublin, Ireland; and Alaris Asena infusion pump, CareFusion UK 306 Ltd, Basingstoke, UK) as well as ambient noise only with all equipment in the room powered down.

The monitor data were downloaded to custom software (Casella Insight version 005-5 data management software; Casella Measurement) and exported to Microsoft Excel (2010 version; Microsoft Corp, Redmond, WA, USA) for analysis. The final graphs were drawn using SigmaPlot version 11 software (Systat Software, Chicago, IL, USA) and Microsoft Excel. Because of their similarity, time and L_Aeq, _data from all units were combined and are presented as averaged L_Aeq _values with confidence limits expressed as ±1.96 SEM.

## Results

Table 1 gives details of the results from the ICUs in the study. The John Radcliffe Hospital Adult ICU is a general adult ICU admitting secondary and tertiary referrals, and the Neurosciences ICU admits patients with neurological or neurosurgical problems and patients after head and neck surgery. The Churchill Hospital Adult ICU is in a hospital without an Emergency Department and admits patients after elective surgery as well as patients from specialist medical and surgical wards. The Royal Berkshire and Wycombe General Hospital ICUs are large-district general hospital ICUs.

**Table 1:** ICU details

Details	John Radcliffe Hospital Adult ICU	Churchill Hospital Adult ICU	John Radcliffe Hospital Neurosciences ICU	Royal Berkshire Hospital ICU	Wycombe General Hospital ICU
Number of beds	12 in 3 bays plus 4 side rooms	6 plus 2 side rooms	12	9 plus 2 side rooms	7 plus 2 side rooms
Nursing handovers	7:30 AM and 7:30 PM	7:30 AM and 7:30 PM	7:30 AM and 7:30 PM	7:30 AM and 7:30 PM	7:30 AM, 1:00 PM and 8:00 PM
Medical handovers	8:30 AM and 8:30 PM	8:30 AM and 8:30 PM	8:30 AM and 8:30 PM	8:00 AM and 8:00 PM	8:00 AM and 8:00 PM
Visiting times	No restrictions	No restrictions	No restrictions	No restrictions (with a quiet period from 3:00 PM to 4:30 PM)	Open visiting except from 1:00 PM to 3:00 PM

**Figure 1:**
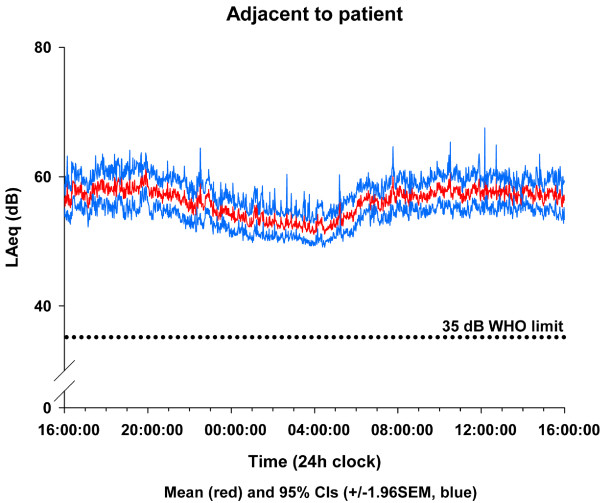
**Average sound levels for patient sited recording device**. Average sound levels at one-minute intervals (L_Aeq_) throughout the day with recording device positioned adjacent to the patient. dB, decibel; WHO, World Health Organisation.

**Figure 2:**
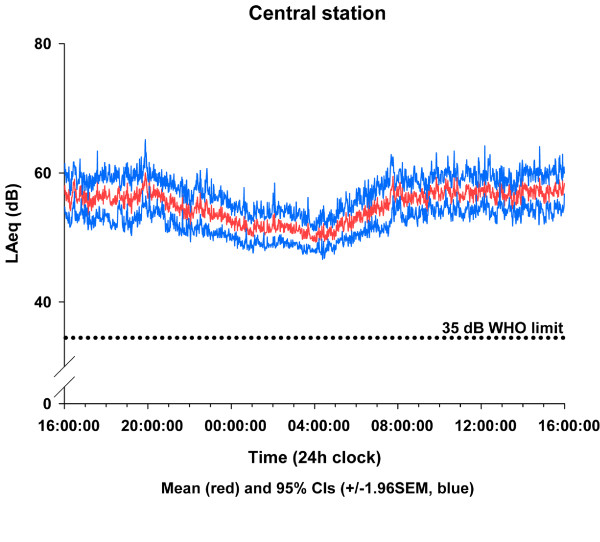
**Average sound levels for centrally sited recording device**. Average sound levels at one-minute intervals (L_Aeq_) throughout the day measured by a recording device on a central station in the ICU. dB, decibel; WHO, World Health Organisation.

Table 2 gives the sound levels averaged for each site over the whole 24-hour period recorded both at the central station and adjacent to the patient. The averaged time × sound-level plots for all ICUs for both recording positions are shown in Figures 1 and 2. All ICUs recorded L_Aeq _levels above 45 dBA at all times and between 52 and 59 dBA for more than 50% of the time.

**Table 2:** Sound levels averaged over 24 hours^a^

Location and day	Central station	Adjacent to patient
John Radcliffe Hospital Adult ICU weekday	58.4 dB	59.7 dB
John Radcliffe Hospital Adult ICU weekend	59.1 dB	59.5 dB
Royal Berkshire Hospital ICU weekday	58.7 dB	59.9 dB
Royal Berkshire Hospital ICU weekend	57.7 dB	58.5 dB
Wycombe General Hospital ICU weekday	52.4 dB	55.4 dB
Wycombe General Hospital ICU weekend	51.3 dB	54.1 dB
John Radcliffe Neurosciences ICU weekday	58.0 dB	58.8 dB
Churchill Hospital Adult ICU weekend	55.7 dB	55.4 dB

^a^dB, decibel. Sound levels averaged over 24 hours for each site. At the John Radcliffe Hospital, the Royal Berkshire Hospital and Wycombe General Hospital, two recordings were measured: on a weekday day and night and on a weekend day and night. Only one recording was taken at the Neurosciences ICU and the Churchill Hospital ICU in Oxford.

**Figure 3:**
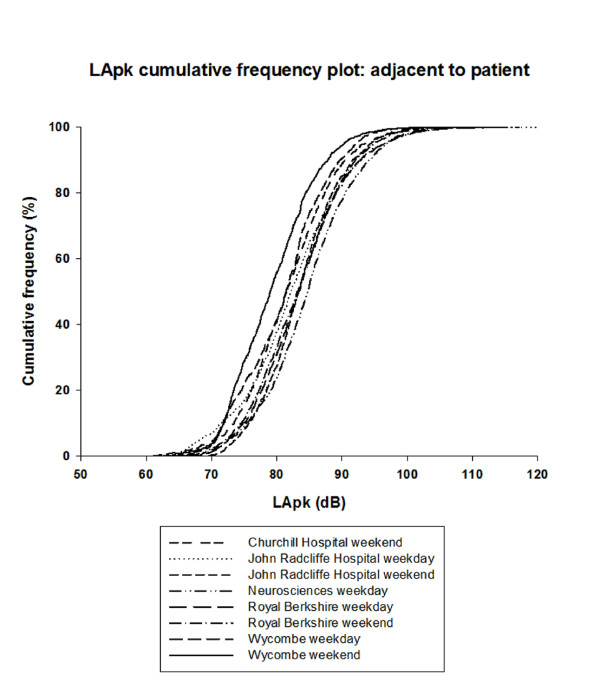
**Peak sound levels for patient-sited recording device**. Peak sound levels (LApk) measured at one-minute intervals throughout the day by a recording device positioned adjacent to the patient. dB, decibel.

Figure 3 gives the cumulative frequency plot of the L_Apeak _recorded minute-by-minute in each ICU adjacent to the patient. In more than 50% of the minutes sampled, L_Apeak _occurred between 79.0 dBA and 84.6 dBA. During every minute sampled, there was a L_Apeak _in excess of 60 dBA. The highest L_Apeak _recorded was 127.9 dBA.

**Figure 4:**
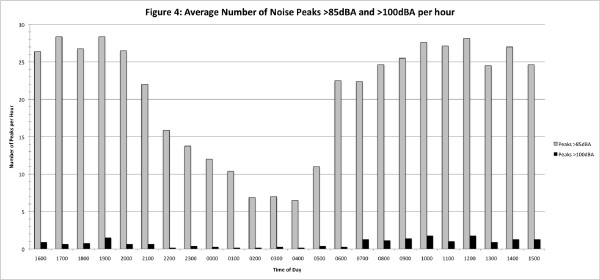
**Average number of peak values per hour for patient-sited recording device**. Average number of minutes per hour when peak values above 85 A-weighted decibels (dBA) and above 100 dBA were recorded with the recording device positioned adjacent to the patient.

Figure 4 shows the average number of minutes per hour across all sites when L_Apeak _above both 85 dBA and 100 dBA were recorded next to the patient. There is a clear diurnal variation that correlates with the L_Aeq _plots of Figures 1 and 2.

**Figure 5:**
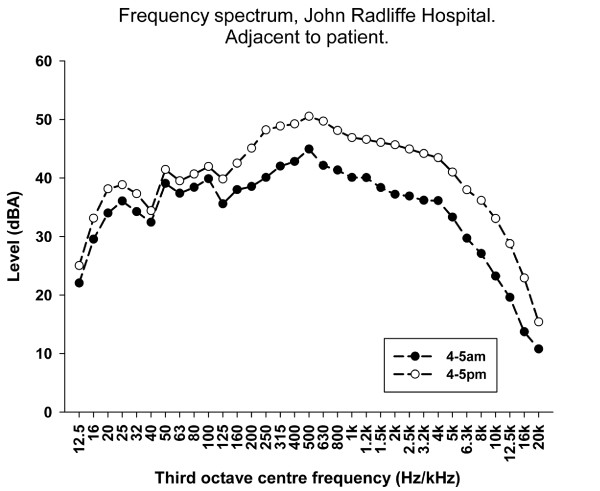
**Frequency plot for 24 hours at John Radcliffe Hospital ICU**. Frequency components of the noise adjacent to a patient at the John Radcliffe Hospital Adult ICU on a weekday at the quietest period (4:00 AM to 5:00 AM) and during working hours (4:00 PM to 5:00 PM).

Figure 5 shows the frequency components of the noise recorded adjacent to a patient in the adult ICU at the John Radcliffe Hospital averaged for an hour at the quietest time (4:00 AM to 5:00 AM) and during working hours (4:00 PM to 5:00 PM). The frequency scale is logarithmic in third-octave frequency bands.

Recordings were also taken at midmorning in an empty side room at the John Radcliffe Hospital Adult ICU with the door closed and the recording equipment placed where the patient's head would normally be situated. With all equipment in the room switched off, the L**_Aeq _**was 34.1 dBA, with increases (1) to 43.5 dBA when the ventilator was running with a test lung; (2) to 47.2 or 51.2 dBA when the ventilator sounded a low- or high-level alert, respectively; (3) to 53.0 dBA with the suction unit turned on; (4) to 59.2 dBA when the monitor sounded a high-level alert; and (5) to 63.3 dBA when the syringe pumps were alerted.

Some frequency components of the alarm noise were identified using one-third octave frequency plots. The physiological monitors showed frequency peaks in the 1.6- to 3.15-kHz bands for the first-level alarm and 2.5- to 3.15-kHz peaks for the second-level (more urgent) alarm. The infusion pump alarms registered frequency peaks in the 800-Hz to 1-kHz range. The ventilator alarms contained a broad spread of frequencies and could not easily be distinguished from other sounds.

## Discussion

Noise is measured using a logarithmic scale of dB. The threshold for normal human hearing is 0 dBA, a quiet room or a whisper is about 30 dBA, normal conversation is about 55 dBA, a television generates about 60 dBA, heavy traffic at 10-m distance is about 80 dBA and a pneumatic drill is about 100 dBA. A 3-dB change in noise level is considered just discernible; a 5-dB change is clearly discernible; and a 10-dB change louder or softer is perceived as a doubling or halving of volume, respectively. For speech to be easily intelligible, it needs to be 15 dB above background noise levels. Thus the recommended WHO average levels for hospital wards are the equivalent of a very quiet room with transient peaks at night well below conversation level.

Although it has been reported that there is no significant reduction in overnight activity in the ICU [5], the link between sleep deprivation and poor outcome has been well-reported in recent years [6-8], and all five units in our present study routinely decrease overnight activity and lower the unit lighting to encourage natural sleeping patterns. The noise levels certainly drop by about 5 dB in the early hours of the morning, but only to the level of continuous conversation. The beginning and end of the night are characterised by obvious increases in noise levels at handover time (see Figures 1 and 2). On average, there were approximately 25 minutes of every hour during the day when peak levels above 85 dBA occurred. Peak levels above 85 dBA occurred less frequently overnight, but a patient can still expect to be disturbed at least once every 7 to 16 minutes of every hour between 10:00 PM and 7:00 AM (Figure 4). At these dB levels, it is highly likely that this is alarm activity, and, as has been reported elsewhere [9], electronic sounds are more arousing than human voices, so they are very likely to continually disturb patients' sleep. Frequent and persistent arousal has been shown to have negative effects for both healthy volunteers and patients [10,11].

Hospitals generally appear to be getting noisier over time. A review of published data over the past 50 years [12] suggests an average increase of 15 dB since the 1960s, more than a doubling of the perceived noise. The same study looked at noise in multiple hospital locations in an American teaching hospital and demonstrated the highest levels in the paediatric ICU, although there were no recordings from the adult ICUs.

It is immediately obvious that sound levels in the Thames Valley Intensive Care Units are considerably higher than the WHO guidelines recommend. The patients are subjected to a continuous level of sound which, at best, is only a little below conversation level and during the day equates to a nearby television or dishwasher. At no point during any of the measurement sessions did the L_Aeq _near the patient fall below 50 dBA. Peak levels (measured as L_Apeak_) were always above 60 dBA and at worst were almost 128 dBA. In previous studies conducted in specialist ICUs, average levels were about 10 dB higher in a Turkish cardiac surgical ICU with a similar time profile [13], similar to our results in a two-bed Swedish neurosurgical ICU with a comparable frequency distribution of peaks [14], and 5 to 10 dB higher in an American paediatric ICU with no diurnal variation [12].

Given the physical and environmental differences in the selected units, it was perhaps surprising that the data generated were so alike. It might be expected that the single-room ICUs would be louder than the three-room John Radcliffe Hospital Adult ICU, but this proved not to be the case. The quietest unit was also not the unit with the lowest number of patients during the recording period. This suggests that noise level is associated with more than simple acoustics and occupancy.

L_Aeq _values were between 51.3 and 59.1 dBA at the central station and 54.1 to 59.9dBA at the patient location. The sound level adjacent to the patient's head was almost always greater than that at the central station. This is probably due to the way equipment is positioned. All the units use pendant or rail systems to suspend the equipment adjacent to the patient's head on each side of the bed, allowing unhindered access to the back of the patient's bed. Whilst this is both convenient and conventional, it does put noise sources close to patients' ears. In all the units studied, the ventilator was positioned at one side of the head of the bed and the monitor was placed on the other side, so these two sources of noise often were 50 cm or less from patients' ears and a similar distance from the recording devices. The noise generated by functioning equipment and alarms seems to be considerable, as evidenced by the levels recorded when the equipment or alarms were recorded in isolation. All units provide patient entertainment (television and/or radio), and it is possible that their use contributed to the slightly higher values recorded at the patient location; however, we did not record television and radio use during the assessment period. We also did not record patient intervention activity, which may have increased noise levels in the bed space.

The frequency components recorded during the day and at the quietest period show a different pattern from that reported in an adult ICU [14] and a paediatric ICU [12] in that there was much less noise below 400 Hz. This is probably simply an effect of the weighting used. We used A-weighting, which is less sensitive to lower frequencies to approximate human perception, which reduces the level at both ends of the frequency spectrum, whereas previously reported results were based on unweighted (absolute sound level) measurements. If the A-weighting is removed, the results more closely resemble the earlier adult ICU results [14]. The change previously noted during the quietest part of the day, with a reduction in sound levels predominantly above 400 Hz [14], was also seen in this study. This may be because much of the lower-frequency sound may be caused by hospital plant and other factors that do not show diurnal variation. By contrast, the higher frequencies, where conversation, alarm sounds and the like are found, do decrease at night. It would be interesting to repeat this exercise at a time of year when the day-night light durations are different to compare seasonal diurnal effects.

A comparable study recorded sound levels in an outpatient chemotherapy clinic [15] and found similar, constant, average (55 to 60 dB) and peak (>90 dB every minute) sound pressure levels during the day. Concurrent questionnaires completed by patients, visitors and staff revealed that, whilst staff felt that the noise was disruptive, in particular causing difficulties with communication, neither patients nor visitors were concerned. Although this suggests that levels seen in the ICU may be acceptable, the authors of that study found a correlation between the time an individual spent at the clinic and the level of irritation expressed. Thus the levels measured in our investigation are likely to affect both staff and patients in the ICU, and attempts should be made to lower noise levels.

The frequency spectra of the alarm sounds were recorded in an attempt to distinguish alarm sounds from background noise, but the acoustic 'signature' of the alarms was difficult to distinguish from the broadband background noise. As a result, we could not consistently measure alarm and non-alarm sounds separately. However, these sounds have been reported elsewhere [5], and it is clear that a significant proportion of the background noise is probably generated from modifiable behaviour such as conversation, operating and moving equipment, telephone use and allowing doors and container lids to close freely. A number of studies have reduced L_Aeq _levels in the adult ICU, at least for a limited period of time, by introducing noise awareness initiatives and unit-level behavioural changes [16-18]. Introducing 'quiet times' has also been shown to improve general well-being [19] and sleep patterns when synchronised with natural circadian rhythms [18].

Three previously reported studies [20-22] used continuous polysomnography alongside environmental noise measurements to determine whether noise could be the reason for irregular sleep patterns in ICU patients and reported that environmental noise caused between 11% and 17% of arousals and awakenings. In interviews after ICU discharge, patients regularly reported disturbed sleep, attributing this to noise, light and frequent nursing interventions [23-28]. Sleep disruption in the ICU is also associated with increased requirements for anxiety and depression treatments [28]. Volunteers exposed to a simulated ICU environment show disturbed sleep and biochemical markers of stress [29], and two studies [25,30] used the ICU Environmental Stressor Scale to assess patient experiences in the ICU. Both studies reported that patients identified alarms as a source of stress. The problem of environmental noise is not limited just to patients; high levels of noise on an ICU have been associated with increased levels of stress for staff [14,31,32]. Studies outside the hospital environment have demonstrated that noise has a negative impact on physiology [33,34], motivation and general health [35].

Mechanical measures to reduce perceived sound levels, such as earplugs or ear defenders, which each reduce perceived noise by 15 to 30 dB, have also been shown to be effective. A recent 136 patient, randomised controlled study in a large Dutch mixed-use ICU showed a dramatic reduction in delirium and an improvement in sleep with this simple intervention [36]. An earlier, smaller US study in a general ICU and a cardiac ICU showed subjectively reported sleep quality was improved with the use of earplugs [37].

Discussions with ICU staff during our data collection period revealed that many we spoke to considered some patient monitor alerts to be disproportionate to their urgency, which led to louder sounds being prolonged while more immediate needs were treated. This inappropriateness in the alarm 'urgency mapping' [38] may quickly lead to desensitisation [39] and a corresponding reduction in alarm response. Alarm fatigue has been cited in a recent report as the leading hazard faced by hospitals in the United States [40]. Visual correlation of the data recorder real-time screens with alarm sounds confirmed that equipment alarms were the likely source of at least some of the peak values. It has been shown that active alarm management can reduce the total number of alarms. A study in the United States [41] introduced a programme by which staff were encouraged to modify machine default limits in line with their patients' individual physiology, thus reducing the opportunity for alarm fatigue to become established. Additionally, the development of smart alarms has been advocated [42-44]. In 2009, Gorges *et al. *[45] reported that only 23% of the alarms in the ICU were 'effective', specifically suggesting that introducing a 19-second delay would eliminate 67% of the ignored and ineffective alarms.

Research is ongoing to improve the system by which patients whose condition is deteriorating are identified [44,46-48], and, although not in widespread use in the United Kingdom, there are alarm management systems which can transfer the audible alert from the patient bedside to a centralised control room or to the care provider. There may therefore be technological solutions that could be used alongside awareness programmes to lower sound levels by more than that which can be achieved by behavioural interventions alone.

We could achieve sound levels within the WHO guidelines only in a closed side room with all patient monitoring equipment switched off. Although some studies have found that it is possible to lower noise levels, at least temporarily, none achieved levels below the WHO guideline limit. Our findings suggest that, with the current equipment required for patient care, the WHO guidelines are not achievable in ICUs in the United Kingdom.

### Limitations

Our study was limited to one day of recording at each site. One full week at each site would have provided more robust data less susceptible to short-term events, which might have affected the sound levels recorded on any given day. We did not collect information on patient sleep assessment or document activity around the patient bed space (for example, treatment and interventions or visitor and/or patient use of television and/or radio), which may have contributed to the noise levels in the patient's vicinity. A more accurate description of the sources of the noise may have been possible with more frequently sampled data, combined with greater frequency discrimination. This would have enabled us to run more detailed analysis of noise levels, particularly with regard to the number of peak levels and their duration.

## Conclusion

The ICUs studied were very noisy places, with a constant level of noise equivalent to that of a lively restaurant. One noise source is obviously staff activity, but ambient noise levels adjacent to the patients are still high, even when this is at a nadir in the early morning, probably due to hospital plant, equipment noise and alarms. Various programmes of staff education, task scheduling, equipment repositioning and alarm threshold review have not lowered sound levels to within WHO-recommended levels. The practical solution within the National Health Service at present seems to be earplugs or other ear defender devices for patients, although there may be opportunities in the future to modulate alerts through the use of smart alarm systems.

## Key messages

• Sound levels in ICUs in the United Kingdom are consistently above WHO-recommended limits.

• L_Apeak _above 60 dBA occurred every minute.

• Earplugs or other ear defender devices can reduce the impact of noise on the patient.

• Environmental changes and/or technological solutions should be investigated as alternatives to noise awareness interventions.

## Abbreviations

dB: decibel; dBA: A-weighted sound level in decibels; L_Aeq_: A-weighted steady noise level containing the same amount of noise energy as in the actual noise, effectively giving an average level over the measurement period (for example, L_Aeq(24 hours)_, L_Aeq(8 hours) _or L_Aeq(day) _and L_Aeq(night)_, where (*n*) refers to the number of hours in the measurement period and (day) and (night) are traditionally understood to be 8:00 AM to 4:00 PM and 4:01 PM to 7:59 PM, respectively); L_Apeak_: maximum level in A-weighted decibels reached by sound pressure at any instant during measurement period (peak is the true peak of the pressure wave, which should not be confused with the highest sound pressure level (L_Amax_); WHO: World Health Organisation.

## Competing interests

The authors declare that they have no competing interests.

## Authors' contributions

Both authors devised the study and collected the data, which were analysed by JDY. JLD wrote the first draft of the manuscript, and JDY made revisions. Both authors read and approved the final version of the manuscript and take equal responsibility for its accuracy.

## Authors' information

Both authors are senior researchers at the Kadoorie Centre Research Group, which is based at the John Radcliffe Hospital in Oxford, UK. JDY is an anaesthetics/intensive care consultant for the Oxford University Hospitals NHS Trust and a Senior Clinical Lecturer at the University of Oxford. JLD is the Critical Care Research Programme Manager for the University of Oxford and has a background in public understanding of science, with a particular interest in the dissemination of research results and the impact research projects have on the patient experience.
